# Body mass index affecting ticagrelor monotherapy vs. ticagrelor with aspirin in patients with acute coronary syndrome: A pre-specified sub-analysis of the TICO randomized trial

**DOI:** 10.3389/fcvm.2023.1128834

**Published:** 2023-03-30

**Authors:** Byung Gyu Kim, Sung-Jin Hong, Byeong-Keuk Kim, Yong-Joon Lee, Seung-Jun Lee, Chul-Min Ahn, Dong-Ho Shin, Jung-Sun Kim, Young-Guk Ko, Donghoon Choi, Myeong-Ki Hong, Yangsoo Jang

**Affiliations:** ^1^ Division of Cardiology, Department of Internal Medicine, Sanggye Paik Hospital, Inje University College of Medicine, Seoul, Republic of Korea; ^2^ Division of Cardiology, Department of Internal Medicine, Severance Cardiovascular Hospital, Yonsei University College of Medicine, Seoul, Republic of Korea; ^3^ Division of Cardiology, Department of Internal Medicine, CHA Bundang Medical Center, CHA University, Seongnam, Republic of Korea

**Keywords:** acute coronary sydrome, body mass index, dual antiplatelet therapy, ticagrelor, drug eluting stent

## Abstract

**Background:**

Although ticagrelor monotherapy after 3-month dual antiplatelet therapy (DAPT) results in a significantly greater net clinical benefit over that with ticagrelor-based 12-month DAPT in patients with acute coronary syndrome (ACS), it remains uncertain whether this effect is dependent on body mass index (BMI). We aimed to evaluate the BMI-dependent effect of these treatment strategies on clinical outcomes.

**Methods:**

This was a pre-specified subgroup analysis from the TICO trial (Ticagrelor Monotherapy After 3 Months in Patients Treated With New Generation Sirolimus-eluting Stent for Acute Coronary Syndrome), evaluating the interaction between BMI and treatment strategies for the primary outcome [composite of major bleeding and adverse cardiac and cerebrovascular events (MACCE): death, myocardial infarction, stent thrombosis, stroke, or target-vessel revascularization]. The secondary outcomes were major bleeding and MACCE.

**Results:**

Based on a pre-specified BMI threshold of 25 kg/m^2^, 3,056 patients were stratified. Patients with BMI <25 kg/m^2^ had a higher risk of primary and secondary outcomes than those with BMI ≥25 kg/m^2^. Regardless of the BMI subgroup, the effects of ticagrelor monotherapy after 3-month DAPT on the primary outcome (*p*_int _= 0.61), major bleeding (*p*_int _= 0.76), and MACCE (*p*_int _= 0.80) were consistent without significant interaction compared with ticagrelor-based 12-month DAPT. The treatment effects according to the BMI quartiles and age, sex, and diabetic status were also consistent without significant interaction.

**Conclusion:**

The BMI-dependent impact of ticagrelor monotherapy after 3-month DAPT compared with 12-month DAPT on clinical outcomes was not heterogeneous in patients with ACS.

**Clinical Trial Registration:**

[www.ClinicalTrials.gov], identifier [NCT02494895].

## Introduction

Body mass index (BMI) is a commonly used obesity indicator and independently affects the cardiovascular outcomes in patients with established coronary artery disease (1–3). Numerous studies have demonstrated that patients with a lower or normal BMI tend to be at a greater risk of ischemic and bleeding events after undergoing percutaneous coronary intervention (PCI) compared with those with a higher BMI (1–3). In addition, BMI can affect the efficacy of dual antiplatelet therapy (DAPT) (4, 5). Therefore, evaluating the efficacy and safety of different post-PCI antiplatelet strategies stratified according to BMI may be important for developing an optimal antiplatelet therapy, which considers individual ischemic and bleeding risks.

Recently, the Ticagrelor Monotherapy After 3 Months in Patients Treated With New Generation Sirolimus-Eluting Stent for Acute Coronary Syndrome (TICO) trial showed the superiority of ticagrelor monotherapy after 3-month DAPT over the currently recommended ticagrelor-based 12-month DAPT in the occurrence of net clinical adverse events in patients with acute coronary syndrome (ACS) (6). However, whether the benefit of this TICO trial strategy is BMI dependent remains unclear. Thus, this pre-specified subgroup analysis of the TICO trial conducted an evaluation for the BMI effect on the efficacy and safety of ticagrelor monotherapy after 3-month DAPT versus ticagrelor-based 12-month DAPT after drug-eluting stent (DES) implantation.

## Materials and methods

### Study design and population

This study is a pre-specified subgroup analysis of the TICO trial. The TICO trial was a multicenter randomized trial investigating the net clinical benefit of ticagrelor monotherapy after 3-month DAPT compared with ticagrelor-based 12-month DAPT in patients with ACS who underwent PCI with ultrathin bioresorbable polymer sirolimus-eluting stents (Orsiro; Biotronik AG, Bülach, Switzerland). Detailed explanations, including inclusion and exclusion criteria, have already been described (6, 7). The trial was approved by the institutional review board at each center and was performed in accordance with the principles of the Declaration of Helsinki. All participants provided written informed consent.

**Figure 1:**
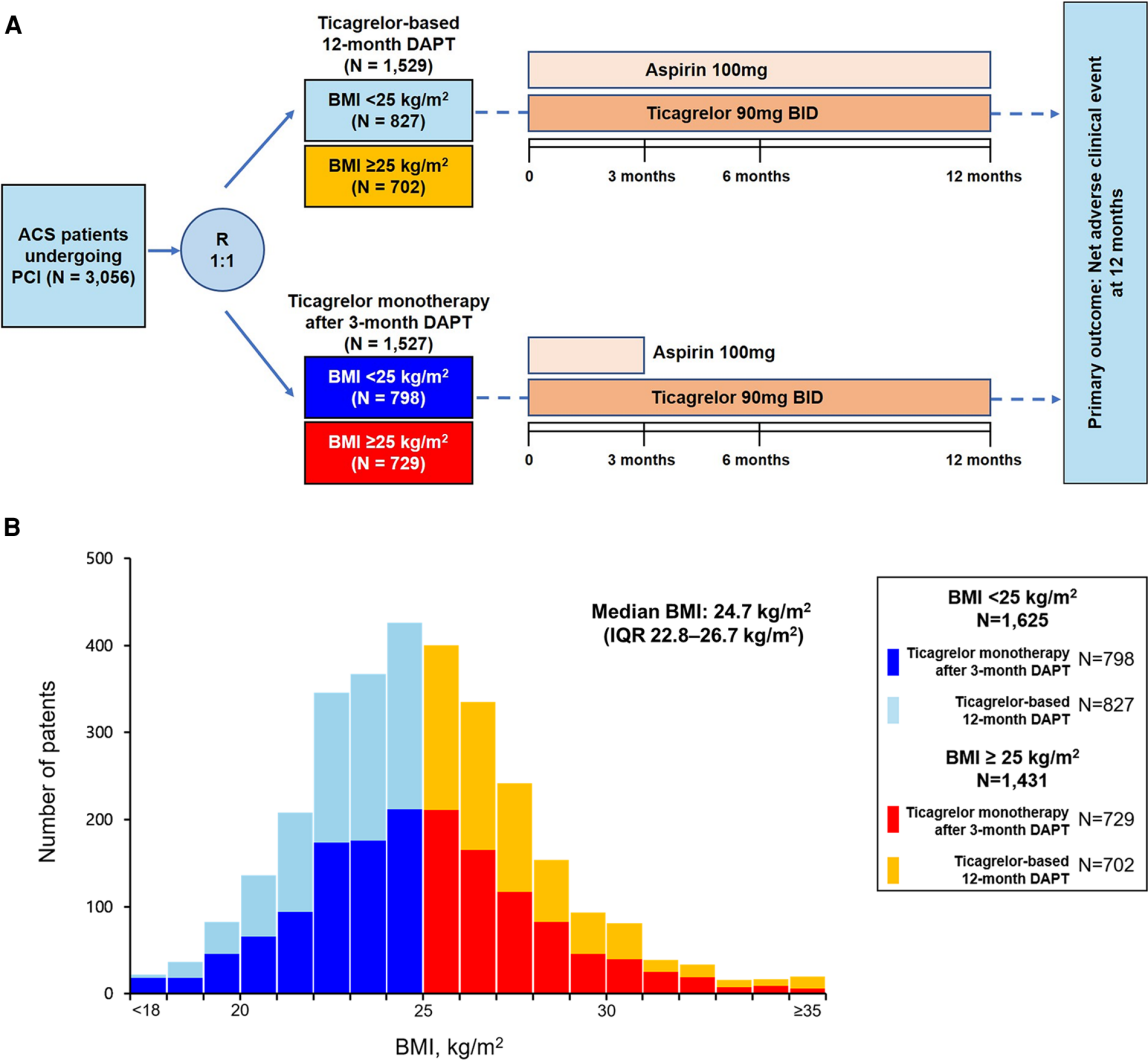
Flowchart of the present study and distribution of BMI. (**A**) Study flow, (**B**) distribution of BMI stratified by antiplatelet therapy strategies. ACS, acute coronary syndrome; BMI, body mass index; DAPT, dual antiplatelet therapy; IQR, interquartile range; PCI, percutaneous coronary intervention.

The TICO trial randomly assigned patients in a 1:1 fashion to receive either ticagrelor monotherapy after 3-month DAPT or ticagrelor-based 12-month DAPT after DES implantation. The baseline BMI was calculated as kg/m^2^ and collected at the time of randomization. For the present investigation on BMI and clinical outcomes, patients were divided into two pre-specified groups: patients with BMI ≥25 and <25 kg/m^2^, respectively (Figure 1A) (6). The Predicting Bleeding Complications in Patients Undergoing Stent Implantation and Subsequent Dual Antiplatelet Therapy (PRCECISE-DAPT) score of each patient was calculated using the online calculator with 5 variables in the database (age, hemoglobin, white blood cell count, creatinine clearance, and previous history of bleeding). The high bleeding risk was defined as the PRECISE-DAPT score ≥25 according to previous study (8).

### Study outcomes

The primary outcome was the occurrence of a net adverse clinical event, defined as a composite of major adverse cardiac and cerebrovascular events (MACCE) and bleeding, 12 months after PCI (6). MACCE was defined as a composite of all-cause death, myocardial infarction, stent thrombosis, stroke, and target-vessel revascularization (6). Major bleeding was defined according to the Thrombolysis in Myocardial Infarction criteria: intracranial bleeding, hemorrhage with at least 5 g/dl decrease in hemoglobin, or fatal bleeding causing death within 7 days (6). The secondary outcomes were major bleeding and MACCE separately. Other individual primary outcome components, cardiac death, major or minor bleeding, and a composite of cardiac death, myocardial infarction, stent thrombosis, or target-vessel revascularization were also analyzed. Additional bleeding endpoints included the bleeding events according to the Bleeding Academic Research Consortium (BARC) criteria type 3 or 5 (9).

### Statistical analysis

BMI-dependent analyses were performed on an intention-to-treat basis. Continuous variables are expressed as mean ± standard deviation and categorical data as frequencies. Baseline and procedural characteristics among the groups were compared using the Student's *t*-test or Mann–Whitney *U* test for continuous variables and Chi-square or Fisher's exact test for categorical variables. Log-rank and Kaplan–Meier tests were used to compare the adverse outcome rates according to antiplatelet strategies. Hazard ratios (HRs) for clinical outcomes were assessed using an unadjusted Cox regression model and are shown with 95% confidence interval (CI). The effect heterogeneity among the subgroups was assessed using interaction terms in the Cox proportional hazard model. Proportional hazards models using restricted cubic splines with 3 knots were developed to explore the association between probability of net adverse clinical event, major bleeding, and MACCE, and BMI as continuous variable and depicted graphically. All tests were two-sided. Statistical significance was set at *p* < 0.05. Statistical analyses were performed using the R Statistical Software (version 3.5.3; R Foundation for Statistical Computing, Vienna, Austria).

## Results

### Baseline characteristics

The BMI distribution among the patients is presented in Figure 1B. The median BMI was 24.7 (interquartile range, 22.8–26.7) kg/m^2^. Density plotting showed no significant difference in the BMI distributions between the two antiplatelet strategy groups (Supplementary Figure S1). Of the 3,056 patients randomized in the TICO trial, 1,625 patients (53.2%) had BMIs <25 kg/m^2^. The baseline characteristics according to the BMI groups and antiplatelet strategies are summarized in Table 1. No significant differences were found in the baseline characteristics according to the antiplatelet strategies in the patients from both the BMI subgroups, except for those who underwent PCI with a transradial approach. However, the baseline characteristics varied according to BMI; patients with BMI <25 kg/m^2^ were older (62.9 ± 10.1 years vs. 58.7 ± 11.0 years; *p* < 0.001), more likely to be female (23.0% vs. 17.8%; *p* = 0.001), had a lower prevalence of hypertension (46.2% vs. 55.2%; *p* < 0.001) or dyslipidemia (56.1% vs. 65.3%; *p* < 0.001), and lower hemoglobin levels (14 ± 1.8 vs. 14.6 ± 1.7 g/dl; *p* < 0.001) and ejection fractions (54.5% vs. 56.7%; *p* = 0.019), and were more frequently to be at a high bleeding risk (19.6% vs. 15.2%; *p* = 0.001) compared with those with BMI ≥25 kg/m^2^ (Table 1 and Supplementary Table S1).

**Table 1:** Baseline characteristics according to the pre-specified BMI subgroups and antiplatelet strategy.

Characteristics	BMI <25 kg/m^2^ (*n* = 1,625)			BMI ≥25 kg/m^2^ (*n* = 1,431)			*p* value*
	Ticagrelor monotherapy after 3-month DAPT (*n* = 798)	Ticagrelor based 12-month DAPT (*n* = 827)	*p* value	Ticagrelor monotherapy after 3-month DAPT (*n* = 729)	Ticagrelor based 12-month DAPT (*n* = 702)	*p* value	
Age, years	62.7 ± 10.1	63.0 ± 10.1	0.519	58.5 ± 11.1	58.9 ± 11.0	0.477	<0.001
Body mass index, kg/m^2^	22.6 ± 1.8	22.6 ± 1.7	0.485	27.5 ± 2.3	27.6 ± 2.7	0.355	<0.001
Female, *n* (%)	189 (23.7)	184 (22.2)	0.530	134 (18.4)	121 (17.2)	0.619	0.001
**Comorbidities, *n* (%)**							
Hypertension	361 (45.2)	390 (47.2)	0.468	399 (54.7)	391 (55.7)	0.754	<0.001
Dyslipidemia	451 (56.5)	460 (55.6)	0.754	473 (64.9)	462 (65.8)	0.754	<0.001
Diabetes	208 (26.1)	215 (26.0)	1.000	210 (28.8)	202 (28.8)	1.000	0.095
Current smoker	275 (34.5)	300 (36.3)	0.476	280 (38.4)	287 (40.9)	0.367	0.017
Chronic kidney disease	158 (19.8)	175 (21.2)	0.536	134 (18.4)	153 (21.8)	0.122	0.799
Prior PCI	67 (8.4)	72 (8.7)	0.893	68 (9.3)	55 (7.8)	0.361	1.000
Prior stroke	28 (3.5)	39 (4.7)	0.272	32 (4.4)	27 (3.8)	0.701	1.000
Prior MI	39 (4.9)	23 (2.8)	0.037	25 (3.4)	26 (3.7)	0.891	0.786
Prior CABG	6 (0.8)	6 (0.7)	1.000	2 (0.3)	4 (0.6)	0.649	0.361
Clinical presentation, *n* (%)			0.140			0.392	0.759
Unstable angina	220 (27.6)	263 (31.8)		222 (30.5)	221 (31.5)		
NSTEMI	284 (35.6)	266 (32.2)		255 (35.0)	222 (31.6)		
STEMI	294 (36.8)	298 (36.0)		252 (34.6)	259 (36.9)		
Hemoglobin, g/dl	14.0 ± 1.8	14.0 ± 1.7	0.690	14.6 ± 1.6	14.6 ± 1.8	0.981	<0.001
Creatinine, mg/dl	1.0 ± 0.8	1.0 ± 0.9	0.210	1.0 ± 0.7	1.1 ± 1.0	0.075	0.714
Ejection fraction, %	54.3 ± 11.8	53.9 ± 12.4	0.564	55.0 ± 11.8	55.3 ± 12. 0	0.709	0.019
PRECISE-DAPT score ≥25, *n* (%)	152 (19.0)	167 (20.2)	0.604	105 (14.4)	112 (16.0)	0.457	0.001
Transradial approach, *n* (%)	443 (55.5)	443 (53.6)	0.461	394 (54.0)	418 (59.5)	0.041	0.232
2- or 3-vessel diseases, *n* (%)	441 (55.3)	467 (56.5)	0.660	401 (55.0)	394 (56.1)	0.710	0.887
Multi-lesion intervention, *n* (%)	163 (20.4)	164 (19.8)	0.812	143 (19.6)	148 (21.1)	0.533	0.920
Total no. of stents per patients	1.4 ± 0.7	1.4 ± 0.7	0.544	1.4 ± 0.7	1.4 ± 0.6	0.490	0.859
Total stent length per patient, mm	34.6 (20.6)	34.8 (21.4)	0.888	34.5 (20.4)	35.2 (19.8)	0.515	0.880
Mean stent diameter, mm	3.2 ± 0.5	3.1 ± 0.4	0.292	3.2 ± 0.4	3.2 ± 0.4	0.274	0.187

Data are presented as mean ± standard deviation or *n* (%). CABG, coronary artery bypass graft; DAPT, dual antiplatelet therapy; MI, myocardial infarction; NSTEMI, non-ST segment elevation myocardial infarction; PCI, percutaneous coronary intervention; PRECISE-DAPT, predicting bleeding complications in patients undergoing stent implantation and subsequent dual antiplatelet therapy; STEMI, ST segment elevation myocardial infarction.

* *p* for the comparison between BMI <25 and ≥25 kg/m^2^.

### Effect of BMI on primary and secondary outcomes

**Figure 2:**
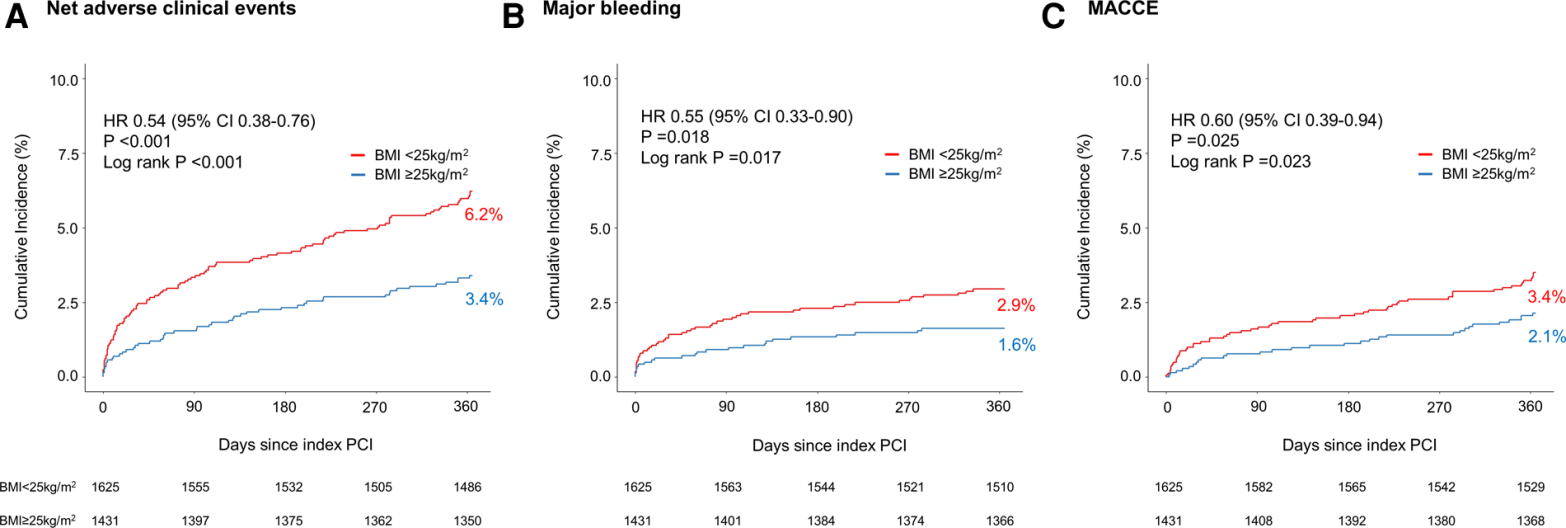
Kaplan–Meier curves for the clinical outcomes between pre-specified BMI subgroups of TICO population. (**A**) Net adverse clinical events, (**B**) major bleeding, and (**C**) major adverse cardiac and cerebrovascular events in patients with either BMI <25 or ≥25 kg/m^2^. BMI, body mass index; CI, confidence interval; HR, hazard ratio; MACCE, major adverse cardiac and cerebrovascular events; PCI, percutaneous coronary intervention.

The primary outcome of the net adverse clinical event occurred more frequently in patients with BMI <25 kg/m^2^ than those with BMI ≥25 kg/m^2^ [100 of 1,625 patients (6.2%) vs. 48 of 1,431 patients (3.4%); *p* < 0.001] (Figure 2A). Regarding the secondary outcomes, major bleeding [47 of 1,625 patients (2.9%) vs. 23 of 1,431 patients (1.6%); *p* = 0.018] and MACCE [56 of 1,625 patients (3.4%) vs. 30 of 1,431 patients (2.1%); *p* = 0.025] occurred more frequently in patients with BMI <25 kg/m^2^ than those with BMI ≥25 kg/m^2^ (Figures 2B,C).

### Effect of antiplatelet strategy on primary and secondary outcomes according to BMI

**Figure 3:**
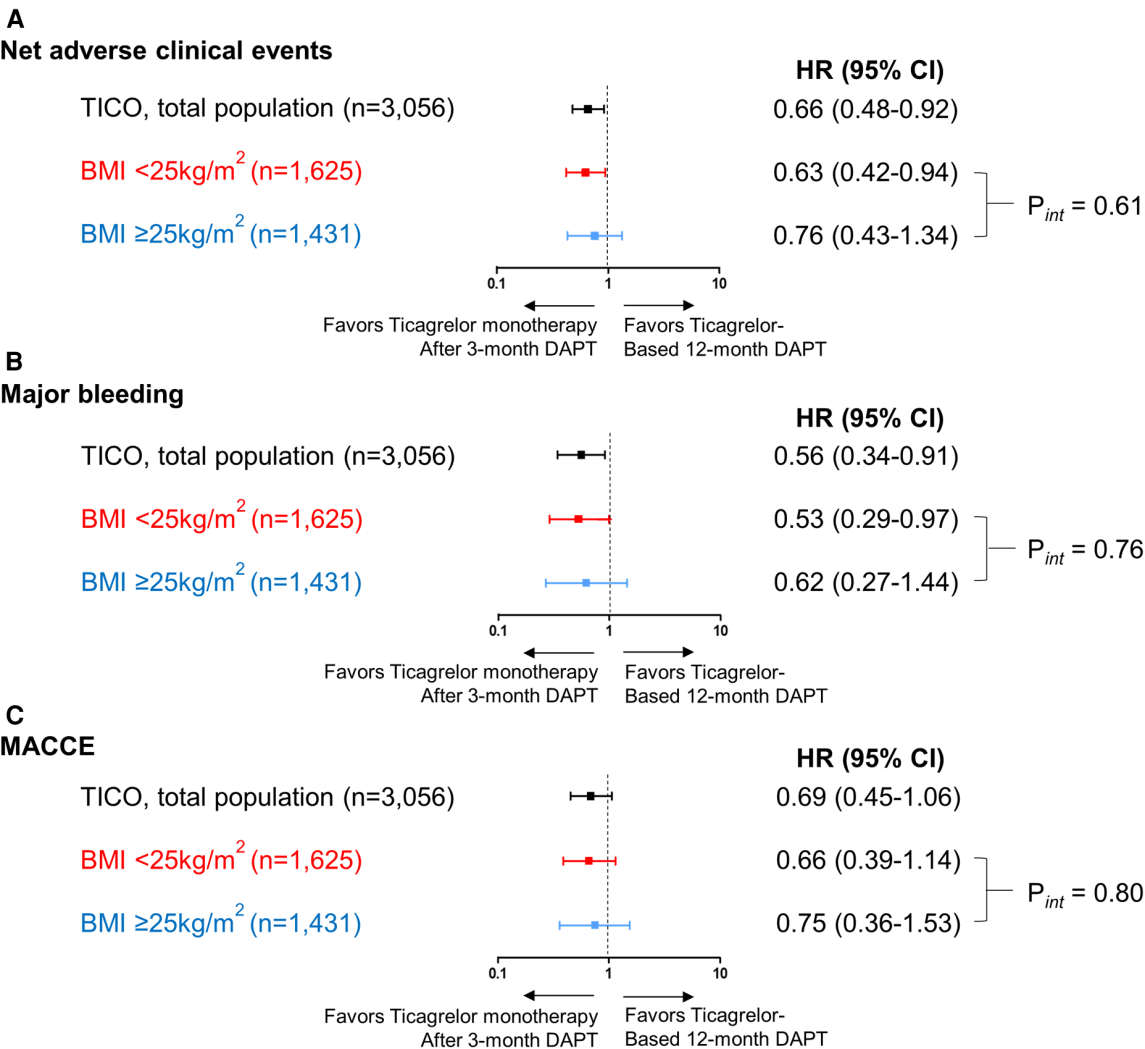
Risk for the primary outcome, major bleeding and MACCE according to the pre-specified BMI subgroups and antiplatelet strategies. Risk for (**A**) net clinical adverse events, (**B**) major bleeding and (**C**) MACCE according to the pre-specified BMI subgroups (BMI <25 and ≥25 kg/m^2^) and antiplatelet strategies. The squares indicate estimated hazard ratio, and the horizontal lines indicate 95% CI. P for interaction values were derived from Cox regression model. BMI, body mass index; CI, confidence interval; DAPT, dual antiplatelet therapy; HR, hazard ratio; MACCE, major adverse cardiac and cerebrovascular events.

In patients with BMI <25 kg/m^2^, ticagrelor monotherapy after 3-month DAPT resulted in a significant primary outcome reduction than ticagrelor-based 12-month DAPT (4.8% vs. 7.5%; HR 0.63; 95% CI 0.42–0.94; *p* = 0.024) (Table 2 and Figure 3A). In patients with BMI ≥25 kg/m^2^, ticagrelor monotherapy after 3-month DAPT compared with ticagrelor-based 12-month DAPT showed a lower primary outcome incidence (2.9% vs. 3.8%; HR 0.76; 95% CI 0.43–1.33; *p* = 0.337) without significant group interaction (*p* for interaction = 0.61) (Table 2 and Figure 3A).

**Table 2:** Clinical outcomes according to the pre-specified BMI groups and antiplatelet strategy at 12 months follow-up.

	BMI <25 kg/m^2^ (*n* = 1,625)				BMI ≥25 kg/m^2^ (*n* = 1,431)				*p* value for interaction
	Ticagrelor monotherapy after 3-month DAPT (*n* = 798)	Ticagrelor-based 12-month DAPT (*n* = 827)	Hazard ratio (95% CI)	*p* value	Ticagrelor monotherapy after 3-month DAPT (*n* = 729)	Ticagrelor-based 12-month DAPT (*n* = 702)	Hazard ratio (95% CI)	*p* value	
**Primary and secondary outcomes**									
Net adverse clinical event	38 (4.8)	62 (7.5)	0.63 (0.42–0.94)	0.024	21 (2.9)	27 (3.8)	0.76 (0.43–1.33)	0.337	0.61
Major bleeding	16 (2.0)	31 (3.7)	0.53 (0.29–0.97)	0.040	9 (1.2)	14 (2.0)	0.62 (0.27–1.44)	0.271	0.76
MACCE	22 (2.8)	34 (4.1)	0.66 (0.39–1.14)	0.135	13 (1.8)	17 (2.4)	0.75 (0.36–1.54)	0.425	0.80
**Individual outcomes**									
Major or minor bleeding	36 (4.5)	58 (7.0)	0.64 (0.42–0.97)	0.034	17 (2.3)	25 (3.6)	0.66 (0.36–1.22)	0.186	0.93
Cardiac death or acute MI	7 (0.9)	17 (2.1)	0.42 (0.18–1.02)	0.056	6 (0.8)	5 (0.7)	1.17 (0.36–3.82)	0.800	0.18
Cardiac death, acute MI, stent thrombosis, or TVR	9 (1.1)	20 (2.4)	0.46 (0.21–1.02)	0.055	6 (0.8)	7 (1.0)	0.83 (0.28–2.48)	0.743	0.39
All-cause death	10 (1.3)	17 (2.1)	0.61 (0.28–1.33)	0.210	6 (0.8)	6 (0.9)	0.97 (0.31–3.02)	0.963	0.50
Cardiac death	3 (0.4)	10 (1.2)	0.31 (0.09–1.13)	0.075	4 (0.5)	2 (0.3)	1.93 (0.35–10.58)	0.445	0.09
Acute MI	4 (0.5)	8 (1.0)	0.51 (0.16–1.71)	0.278	2 (0.2)	3 (0.4)	0.65 (0.11–3.89)	0.636	0.83
Stent thrombosis	2 (0.3)	4 (0.5)	0.52 (0.09–2.82)	0.445	4 (0.5)	0	-	-	1.00
Stroke	4 (0.5)	7 (0.8)	0.59 (0.17–2.01)	0.398	4 (0.5)	4 (0.6)	0.98 (0.24–3.90)	0.972	0.60
TVR	4 (0.5)	4 (0.5)	1.03 (0.26–4.11)	0.968	0	2 (0.3)	-	-	1.00
BARC 3 or 5 bleeding	36 (4.5)	58 (7.0)	0.64 (0.42–0.97)	0.034	17 (2.3)	25 (3.6)	0.66 (0.36–1.22)	0.186	0.93

BARC, bleeding academic research consortium; BMI, body mass index; CI, confidence interval; DAPT, dual antiplatelet therapy; MACCE, major adverse cardiac and cerebrovascular events; MI, myocardial infarction; TVR, target-vessel revascularization.

Ticagrelor monotherapy after 3-month DAPT showed a lower incidence of major bleeding compared with that of ticagrelor-based 12-month DAPT in patients with BMI <25 kg/m^2^ (Table 2 and Figure 3B). In patients with BMI ≥25 kg/m^2^, fewer bleeding events occurred following ticagrelor monotherapy after 3-month DAPT than ticagrelor-based 12-month DAPT without statistical significance. The occurrences of MACCE with the two antiplatelet strategies were not significantly different in both the BMI subsets (Table 2 and Figure 3C). There were no interactions between the BMI subgroup and antiplatelet strategy for major bleeding or MACCE (*p* for interaction = 0.76 or 0.80, respectively).

**Figure 4:**
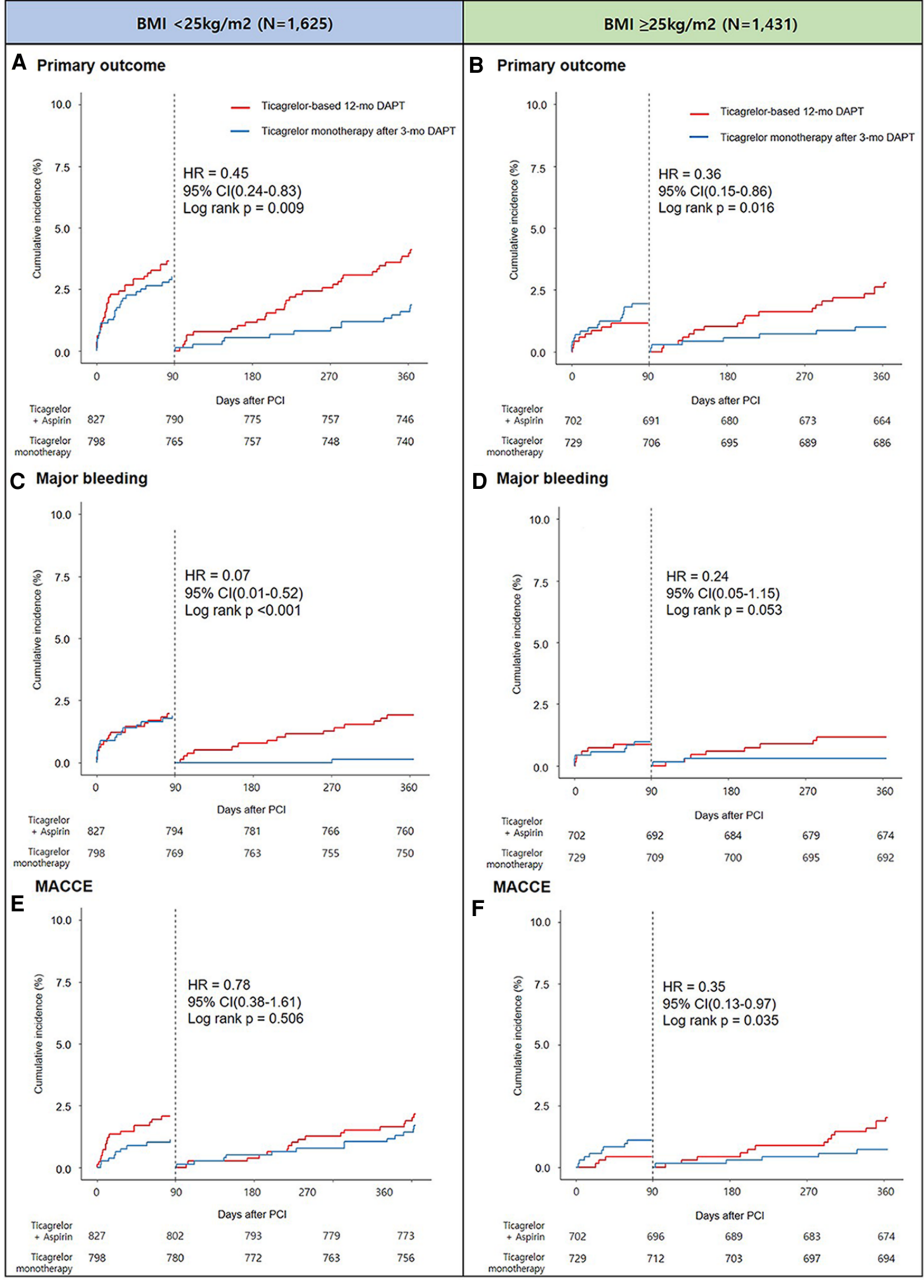
Landmark analysis at 3 months and Kaplan-Meier estimates for primary outcome in patients with BMI <25 kg/m^2^ (**A**) and BMI ≥25 kg/m^2^ (**B**), major bleeding in patients with BMI <25 kg/m^2^ (**C**) and BMI ≥25 kg/m^2^ (**D**), and MACCE in patients with BMI <25 kg/m^2^ (**E**) and BMI ≥25 kg/m^2^ (**F**).

The pre-specified 3-month landmark analysis revealed that regardless of BMI <25 or ≥25 kg/m^2^, the primary outcome incidence was significantly lower in the ticagrelor monotherapy after 3-month DAPT group than in the ticagrelor-based 12-month DAPT group (Figures 4A,B). Similar trends of a lower incidence of major bleeding (Figures 4C,D) and MACCE (Figures 4E,F) in the ticagrelor monotherapy after 3-month DAPT group than in the ticagrelor-based 12-month DAPT group were observed, regardless of the BMI subgroups. Treatment effect analysis according to the BMI quartiles (Q1 to Q4) revealed that the effect of ticagrelor monotherapy after 3-month DAPT was remarkable in patients with BMI quartile 2; however, there was no significant interaction between the BMI quartiles and treatment effects (*p* for interaction = 0.79) (Supplementary Figure S2). When examined as a continuous variable, there were no significant interactions between the antiplatelet strategies and BMI in terms of net clinical adverse event, major bleeding, and MACCE (Supplementary Figure S3).

### Effect of antiplatelet strategies on primary outcome according to BMI and other covariates, such as age, sex, and diabetes

Interactions between the BMI groups and treatment strategies for the primary outcome according to covariates, such as age, sex, and diabetes, are shown in Supplementary Figure S4. There were no between-group differences in the net adverse clinical events of the two antiplatelet strategies, regardless of the age group (<65 years, *p* for interaction = 0.16; ≥65 years, *p* for interaction = 0.28), sex (men, *p* for interaction = 0.72; women, *p* for interaction = 0.18), or diabetic status (diabetic, *p* for interaction = 0.55; non-diabetic, *p* for interaction = 0.93).

## Discussion

In this pre-specified sub-analysis from the TICO trial, we found that: (i) the occurrences of the primary outcome, major bleeding, and MACCE were significantly higher in patients with ACS with low (<25 kg/m^2^) rather than high (≥25 kg/m^2^) BMIs; (ii) there were no significant interactions between the antiplatelet strategies and BMI subgroups for the primary outcome, major bleeding, and MACCE, which suggest a consistent BMI-independent effect of ticagrelor monotherapy after 3-month DAPT compared with that of ticagrelor-based 12-month DAPT; and (iii) the effect of ticagrelor monotherapy was consistent across the BMI subgroups, despite other major covariates, such as age (<65 or ≥65 years), sex (men or women), and diabetic status.

BMI may affect the occurrence of ischemic or bleeding. A higher BMI, such as that indicating obesity, is a well-known cardiometabolic risk factor and could be associated with a highly prothrombotic condition due to the increased risk of impaired endothelial function, platelet activation *via* enhanced oxidative stress, and consequent proinflammatory status, which may accelerate thrombogenicity by increasing the intrinsic platelet reactivity (10, 11). However, several studies have reported a paradoxical association between higher BMI and lower incidence of adverse outcomes, including ischemic events and mortality, called “obesity paradox” (2, 3, 12). The present study was congruent with these previous ones, and demonstrated that patients with low BMI (<25 kg/m^2^) had a greater risk of ischemic events than those with high BMI (≥25 kg/m^2^). Patients with a lower BMI who underwent PCI tend to be older, more fragile, and have more frequent renal insufficiency episodes compared with those with a higher BMI (3), which could increase the bleeding risk. Additionally, an association between low BMI and increased post-PCI bleeding risk has been reported in previous observational studies (13, 14), and this consistent association was observed in our study. Therefore, the BMI-dependent effect, especially on a novel antiplatelet therapy after DES implantation needs to be further assessed.

Pharmacokinetic parameters or metabolic regulation of drugs can be altered based on the BMI (15). According to previous observational studies, high BMI was an independent predictor of high residual platelet reactivity with DAPT containing clopidogrel (16, 17). The beneficial effect of ticagrelor compared with clopidogrel was remarkable when body weight was higher than the median value for each sex in the PLATO trial (18). Another study revealed that the inhibition of platelet aggregation after a high loading dose of 600 mg of clopidogrel was less effective in overweight patients compared with normal-weight patients (19), suggesting that the effectiveness of antiplatelet strategies might vary according to the BMI.

In this study, there was no significant interaction between the ticagrelor-based short DAPT strategy and BMI subgroups for the primary and secondary ischemic or bleeding outcomes, which can be attributed to the following reasons. Unlike clopidogrel, ticagrelor is an active metabolite and does not require metabolic activation in the liver (20); therefore, it may be less affected by BMI and prevent the increased residual platelet reactivity observed with clopidogrel use in overweight patients, even after early aspirin discontinuation (16). Although few studies have reported the effects of ticagrelor according to BMI, Nardin et al. reported that there was no evidence that BMI had a significant influence on the effectiveness of ticagrelor maintenance treatment (16). In addition, regarding the DAPT duration with a potent P2Y12 inhibitor regimen, the present subgroup analysis of the TICO trial found that ticagrelor monotherapy after 3-month DAPT showed a consistently beneficial effect in terms of net clinical benefits compared with that of ticagrelor-based 12-month DAPT in patients with both high and low BMIs. Our results suggest that there may be a low attenuation risk of the ticagrelor effect with increasing BMI, and that an aspirin-free strategy can safely decrease the bleeding risk irrespective of BMI. Thus, ticagrelor monotherapy after short-term DAPT might be a safe and effective strategy in patients with ACS after PCI across the BMI groups.

## Study limitations

Our study had some limitations. First, although this was a pre-specified subgroup analysis, the BMI subgroups were not specifically powered for the occurrence of the primary or secondary outcomes. Thus, the results obtained in these subgroups should be considered as hypothesis-generating results. Second, because this study was conducted based on the baseline BMI data, the effects of BMI fluctuation or changes during the follow-up period were not evaluated. Third, a BMI cut-off value of 25 kg/m^2^ was used for this analysis, which is the cut-off for distinguishing normal weight and overweight according to the World Health Organization standards. In addition, our threshold was close to the median value of 24.7 kg/m^2^, which allows uniform statistical power in the BMI groups. Fourth, our study population comprised a homogeneous Korean ethnicity. Because of anthropometric differences, including body physique and BMI, between eastern and western populations, generalization of our results for other ethnic populations should be performed with caution. Fifth, since the platelet function test was not routinely performed in our cohort, pharmacodynamic data responses to antiplatelet therapy was not available in this study. Given the fact that obesity was found to be an independent predictor of high residual platelet reactivity to DAPT in previous study, further studies on platelet function-guided antiplatelet therapy in cohort involving a larger number of obese patients are needed. Finally, since the TICO trial was conducted exclusively in patients who underwent ultrathin sirolimus-eluting stent implantation, generalization of our results for populations treated with other DESs should be undertaken with caution.

## Conclusion

Overall, the benefit of ticagrelor monotherapy after 3-month DAPT over ticagrelor-based 12-month DAPT on net adverse clinical events was uniform across the BMI subgroups in patients with ACS who underwent ultrathin sirolimus-eluting stent implantation. Treatment effects with respect to major bleeding and MACCE were also similar in the BMI subsets. These findings suggest that ticagrelor monotherapy after 3-month DAPT may be an attractive and safe alternative treatment for patients with ACS, regardless of their BMIs.

## Data Availability

The datasets generated for the analyses are not publicly available because of strict government restrictions. Requests to access these datasets should be directed to B-K Kim, kimbk@yuhs.ac.
