# Supplementary material for: Body mass index affecting ticagrelor monotherapy vs. ticagrelor with aspirin in patients with acute coronary syndrome: A pre-specified sub-analysis of the TICO randomized trial
Source: Front Cardiovasc Med. 2023 Mar 30;10:1128834. doi: 10.3389/fcvm.2023.1128834 (PMC10098124; doi:10.3389/fcvm.2023.1128834)
Supplement: Supplementary file 1 [file Datasheet1.docx]

**Supplementary Materials**

Kim BG, et al. Body Mass Index and Outcomes Following Ticagrelor Monotherapy vs Ticagrelor With Aspirin in Acute Coronary Syndrome Patients Undergoing Percutaneous Coronary Intervention: a prespecified sub-analysis of the TICO randomized trial

**Contents**

I. Supplementary Figure 1. Density plot representing the distribution of body mass index according to antiplatelet strategies.

II. Supplementary Table 1. Baseline characteristics according to pre-specified body mass index subgroups.

III. Supplementary Figure 2. Risks of clinical outcomes according to body mass index quartile and antiplatelet strategies

IV. Supplementary Figure 3. Effect of ticagrelor monotherapy versus dual antiplatelet therapy according to the body mass index

V. Supplementary Figure 4 Effect of antiplatelet strategies according to body mass index and other covariate such as age, sex, and diabetes status

**Supplementary Figure 1. Density plot representing the distribution of body mass index according to antiplatelet strategies.**

**
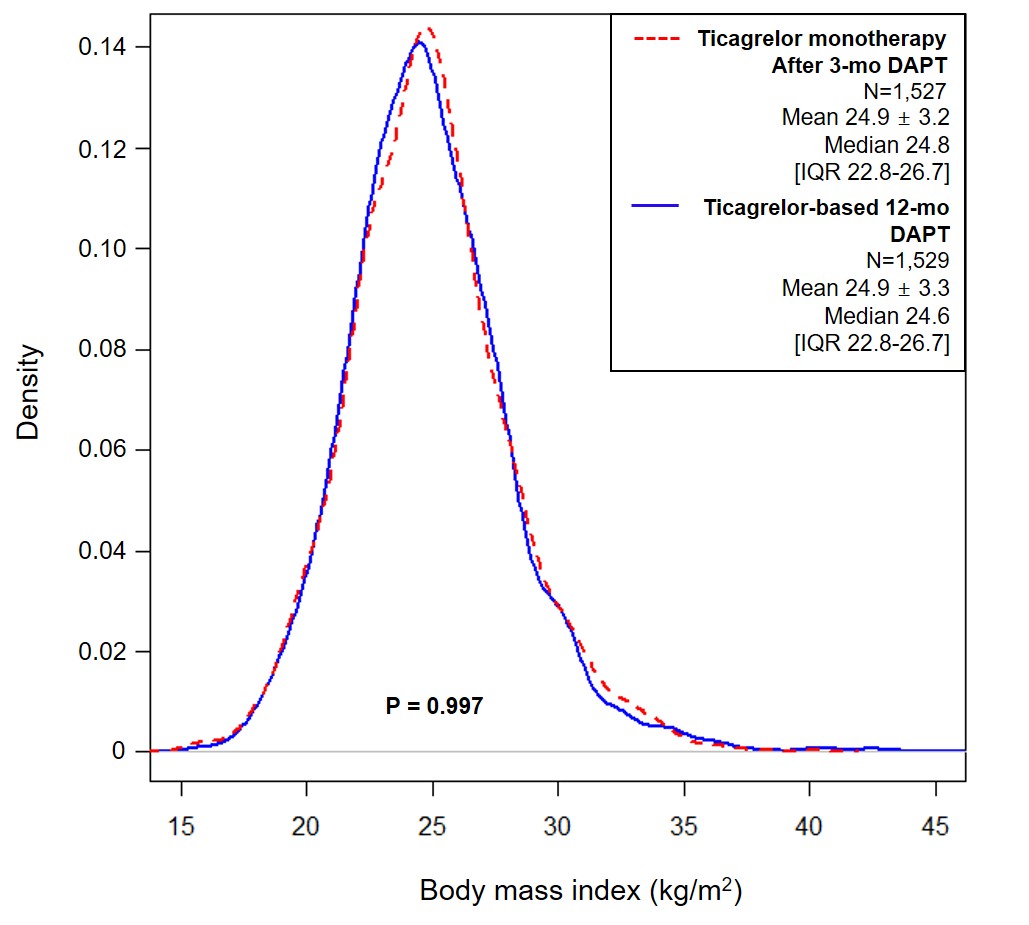
**

**Supplementary Table 1. Baseline characteristics according to pre-specified body mass index subgroups.**

| Characteristics | BMI <25 kg/m^2^ (n = 1,625) | BMI ≥25 kg/m^2^ (n = 1,431) | P value |
| --- | --- | --- | --- |
| Age, y | 62.9 ± 10.1 | 58.7 ± 11.0 | <0.001 |
| BMI, kg/m^2^ | 22.6 ± 1.7 | 27.5 ± 2.5 | <0.001 |
| Female | 373 (23.0) | 255 (17.8) | 0.001 |
| Comorbidities, n (%) |  |  |  |
| Hypertension | 751 (46.2) | 790 (55.2) | <0.001 |
| Dyslipidemia | 911 (56.1) | 935 (65.3) | <0.001 |
| Diabetes | 423 (26.0) | 412 (28.8) | 0.095 |
| Current smoker | 575 (35.4) | 567 (39.6) | 0.017 |
| Chronic kidney disease | 333 (20.5) | 287 (20.1) | 0.799 |
| Prior PCI | 139 (8.6) | 123 (8.6) | 1.000 |
| Prior stroke | 67 (4.1) | 59 (4.1) | 1.000 |
| Prior MI | 62 (3.8) | 51 (3.6) | 0.786 |
| Prior CABG | 12 (0.7) | 6 (0.4) | 0.361 |
| Clinical presentation, n (%) |  |  | 0.759 |
| Unstable angina | 483 (29.7) | 443 (31.0) |  |
| NSTEMI | 550 (33.8) | 477 (33.3) |  |
| STEMI | 592 (36.4) | 511 (35.7) |  |
| Hemoglobin, g/dL | 14 ± 1.8 | 14.6 ± 1.7 | <0.001 |
| Creatinine, mg/dL | 1.0 ± 0.8 | 1.0 ± 0.8 | 0.714 |
| Ejection Fraction, % | 54.1 ± 12.1 | 55.1 ± 11.9 | 0.019 |
| PRECISE-DAPT score ≥25, n (%) | 319 (19.6) | 217 (15.2) | 0.001 |
| Transradial approach, n (%) | 886 (54.5) | 812 (56.7) | 0.232 |
| Multi-vessel diseases, n (%) | 908 (55.9) | 795 (55.6) | 0.887 |
| Multi-lesion intervention, n (%) | 327 (20.1) | 291 (20.3) | 0.920 |
| Total No. of stents per patients | 1.4 ± 0.7 | 1.4 ± 0.7 | 0.859 |
| Total stent length per patient, mm | 34.7 ± 21.0 | 34.8 ± 20.1 | 0.880 |
| Mean stent diameter, mm | 3.1 ± 0.4 | 3.2 ± 0.4 | 0.187 |

BMI = Body mass index; CABG = coronary artery bypass graft; DAPT = dual antiplatelet therapy; MI = myocardial infarction; NSTEMI = non-ST segment elevation myocardial infarction; PCI = percutaneous coronary intervention; PRECISE-DAPT = predicting bleeding complications in patients undergoing stent implantation and subsequent dual antiplatelet therapy; STEMI = ST segment elevation myocardial infarction

**Supplementary Figure 2. Risks of clinical outcomes according to body mass index** **quartile and antiplatelet strategies**

Risks of (A) net adverse clinical events, (B) major bleeding, and (C) MACCE. Hazard ratios are for patients administered ticagrelor monotherapy after 3-month of DAPT vs. ticagrelor-based 12-month DAPT (BMI Q1 = 15.4 to 22.7 kg/m^2^; BMI Q2 = 22.8 to 24.6 kg/m^2^; BMI Q3 = 24.7 to 26.6 kg/m^2^; and BMI Q4 = 26.7 to 48.2 kg/m^2^). BMI = body mass index; CI = confidence interval; DAPT = dual antiplatelet therapy; MACCE = major adverse cardiac and cerebrovascular event


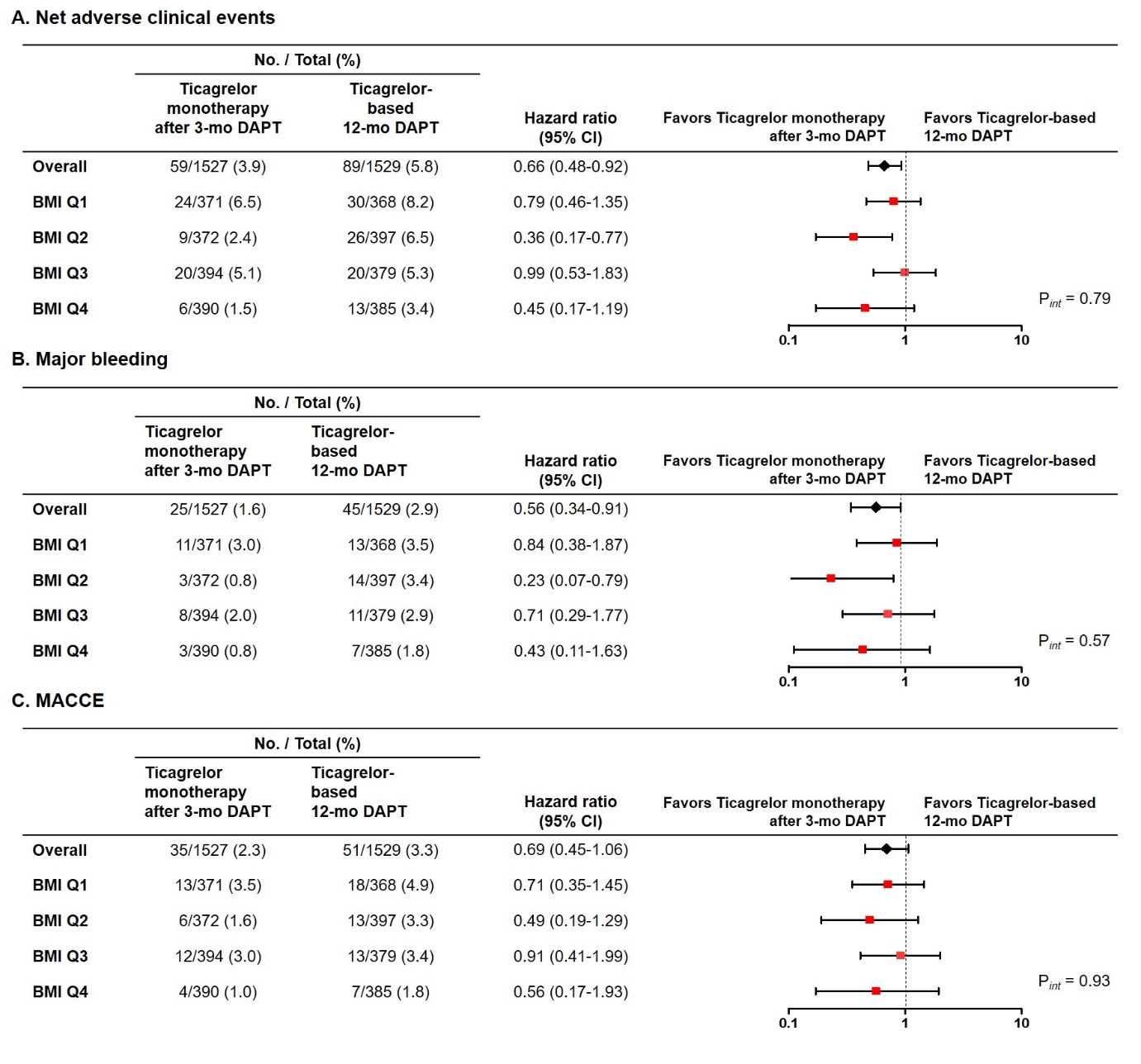


**Supplementary Figure 3. Effect of ticagrelor monotherapy versus dual antiplatelet therapy according to the body mass index.**

There were no significant interactions between the antiplatelet strategies and body mass index in terms of net adverse clinical event, major bleeding, and MACCE. HR = hazard ratio; MACCE = major adverse cardiac and cerebrovascular event


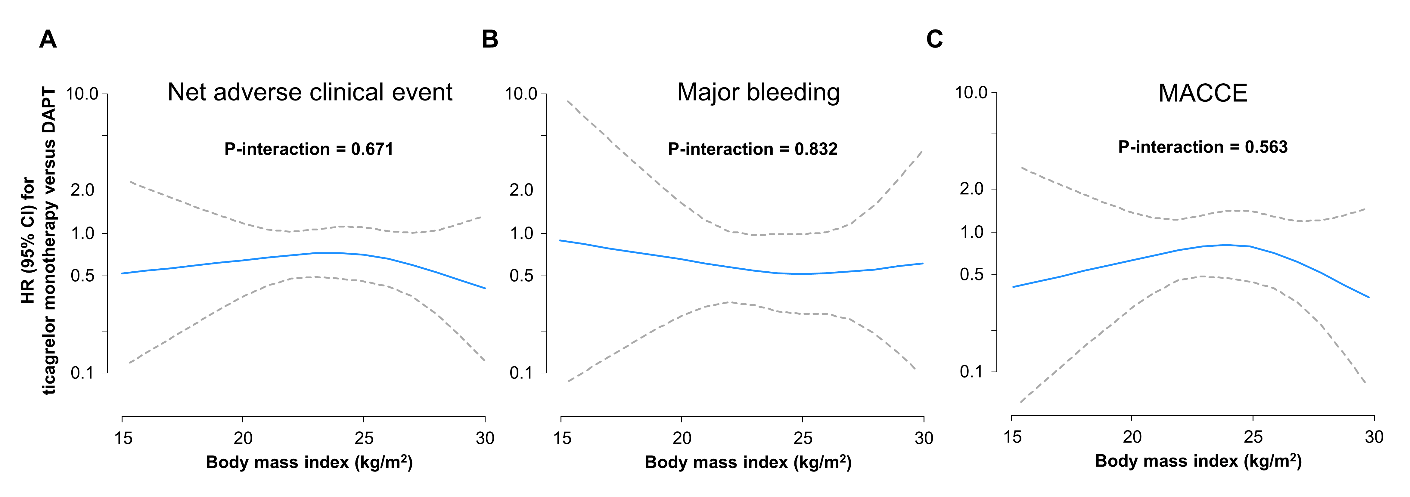


**Supplementary Figure 4. Effect of antiplatelet strategies according to body mass index and other covariate such as age, sex, and diabetes status**

Hazard ratios are for patients administered ticagrelor monotherapy after 3-month of DAPT vs. ticagrelor-based 12-month DAPT. BMI = body mass index; CI = confidence interval; DAPT = dual antiplatelet therapy

**
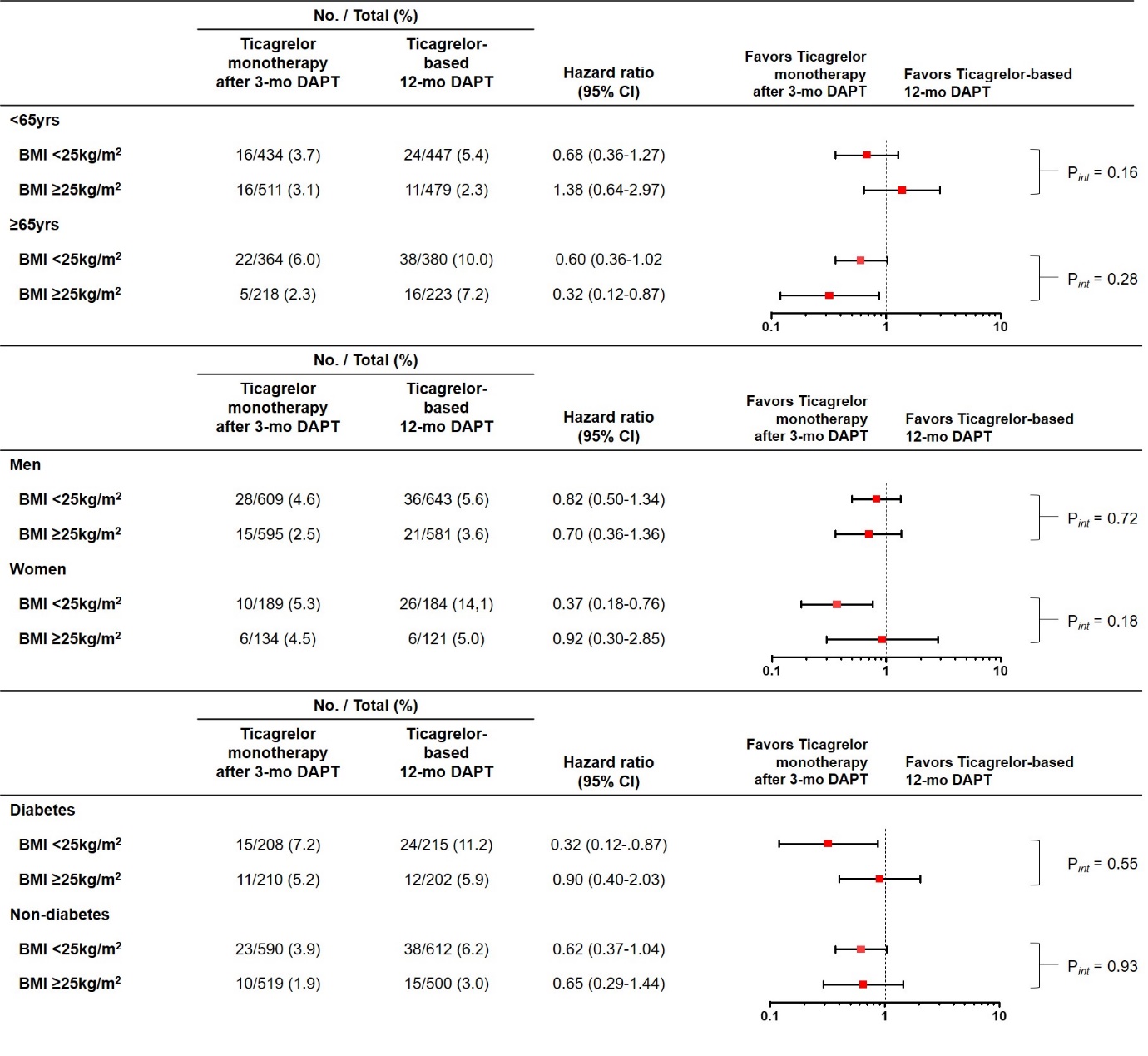
**
